# Semiconductor-nanoantenna-assisted solar absorber for ultra-broadband light trapping

**DOI:** 10.1186/s11671-020-03311-2

**Published:** 2020-04-08

**Authors:** Yuyin Li, Zhengqi Liu, Pingping Pan, Xiaoshan Liu, Guolan Fu, Zhongmin Liu, Haimei Luo, Guiqiang Liu

**Affiliations:** Jiangxi Key Laboratory of Nanomaterials and Sensors, School of Physics, Communication and Electronics, Jiangxi Normal University, Nanchang, 330022 China

**Keywords:** Solar absorbers, Nanoantennas, Short-circuit current density, Light trapping

## Abstract

Light trapping is an important performance of ultra-thin solar cells because it cannot only increase the optical absorption in the photoactive region but it also allows for the efficient absorption with very little materials. Semiconductor-nanoantenna has the ability to enhance light trapping and raise the transfer efficiency of solar energy. In this work, we present a solar absorber based on the gallium arsenide (GaAs) nanoantennas. Near-perfect light absorption (above 90%) is achieved in the wavelength which ranges from 468 to 2870 nm, showing an ultra-broadband and near-unity light trapping for the sun’s radiation. A high short-circuit current density up to 61.947 mA/cm^2^ is obtained. Moreover, the solar absorber is with good structural stability and high temperature tolerance. These offer new perspectives for achieving ultra-compact efficient photovoltaic cells and thermal emitters.

## Introduction

Solar energy, as a renewable, clean, and widespread energy, is widely studied because it can be transformed into other energies for wide applications such as solar cells [1–3], photovoltaic devices [4, 5], and photo-thermal emitters [6, 7]. Since Landy et al. reported the perfect absorbers based on the metal-insulator-metal triple-layer meta-materials [8], a plenty of fascinating nanostructures have been designed for the collection and utilization of solar energy [9–21]. It is worth noting that the efficient solar energy capture is a key for these applications. Therefore, the solar absorption response of the absorbers is usually studied to evaluate the performance of solar energy collection. The ideal absorber possesses near-unity absorption in a wide wavelength range.

In principle, the perfect absorber means a good thermal emitter in the same wavelength range. For a given temperature, the energy of radiation can be well described and detected by the absorption of the structure [7]. Moreover, the absorption ratio to the thermal radiation is always equal to the emissivity under the thermal equilibrium conditions. Noble metallic nanostructures are usually utilized to obtain perfect absorbers, extraordinary light transmission or Fano resonances via strong coupling of light with surface plasmons [22–30]. However, the absorbed solar energy would lead to the increase in temperature (i.e., thermal instability), resulting in the damage of noble metallic nanostructures with low melting point [7]. Note that the structural stability and high temperature tolerance can be guaranteed when refractory metals are used to replace noble metals in the absorbers [6, 9, 11, 12]. Although the broadband light absorption phenomena were demonstrated in these platforms, these methods may suffer from problems such as the sophisticated geometries [6, 18], relatively finite absorption bandwidths (< 750 nm) [9, 11, 12], or large requirement of noble metals [8, 10, 11, 18].

Semiconductor materials have also drawn intensive interest due to their low cost and high conversion efficiency for solar energy as compared with the conventional thin-film devices [31–39]. Most of the solar absorbers are based on silicon (Si) due to its natural abundance and nearly ideal energy band gap [31, 34]. However, the efficiency of solar cells is limited when the thickness of Si layers reduces. Therefore, light trapping has now become one of the major topics in the thin film solar cells [38]. Recently, gallium arsenide (GaAs) has become a good competitor because of its unique optical property and high conversion efficiency [36–39], which have been demonstrated experimentally in solar harvesting. For instance, Massiot et al. presented the metal nanogrid for broadband multi-resonant light-harvesting in the ultrathin GaAs layers with the absorption bandwidth of 380 nm (from 450 to 830 nm) [40]. Li et al. proposed a solar cell by combining gold nanoparticles and GaAs nanowire arrays to realize the wide absorption band in the visible region (300–850 nm) [39]. However, their absorption bands are almost within the range of 300–1100 nm. Recently, by placing the GaAs grating on a GaAs-tungsten (W) bi-layer-film structure, we obtained a perfect absorber [40]. However, the absorption (> 90%) bandwidth only reaches 1300 nm. Furthermore, only the transverse-magnetic (TM) polarization is considered in this structure.

In this work, we propose a feasible solar absorber based on the semiconductor GaAs and refractory metals W and Ti. A one-dimensional (1D) GaAs nanoantenna period array, coated by the indium tin oxide (ITO) antireflection (AR) nanoantennas, is placed on the thin W-GaAs-Ti three-layer film structure. This solar absorber presents an ultra-broad absorption band spanning the visible and middle infrared regions due to the synergy of guide mode resonances (GMRs) and cavity resonant modes together with surface plasmon polaritons (SPPs). The bandwidth with the absorption over 90% is larger than 2400 nm. The absorber also shows a good tolerance to the angle and polarization of incident light. In addition, high short-circuit current density up to 61.947 mA/cm^2^ is achieved under the AM1.5 solar illumination. These offer new perspectives for achieving ultra-compact efficient photovoltaic cells and thermal emitters.

## Materials and Method

**Fig. 1:**
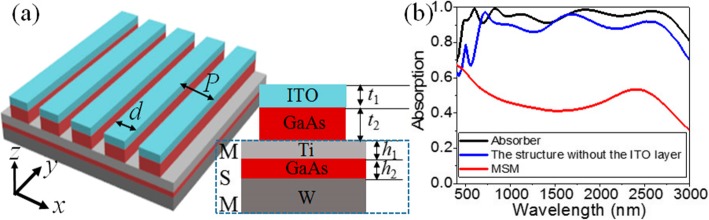
**a** Schematic of the proposed solar absorber. **b** Absorption spectra of the solar absorber (black line), MSM stack structure (red line), and MSM structure coated with only GaAs nanoantennas (blue line)

The schematic of the proposed absorber is shown in Fig. 1a. A 1D GaAs nanoantenna array is sandwiched by a single-layer AR array made of ITO nanoantennas and a thin metal-semiconductor-metal (MSM) three-layer film structure. Although noble metals are indispensable in creating broadband absorption structures, they suffer from low melting points [41]. In addition, due to the small-size effect, the melting points of patterned noble metal nanostructures are greatly reduced [42]. These lead to noble metallic nanostructures not meeting with the working temperature of solar photovoltaics. Therefore, the materials with ultra-high thermal stability and light absorption capacity are highly desired to keep the stability of solar absorbers. Metallic W, titanium (Ti) [6, 17], and semiconductor GaAs [36, 37, 39] are all with high melting points (3422 °C, 1668 °C, and 1238 °C at room temperature, respectively) and thus are employed to obtain ultra-broad absorption bands in this work. The period and width of the nanoantennas are denoted as *P* and *d*, respectively. The thickness of the bottom W film is 100 nm. The thicknesses of Ti and GaAs films are respectively marked with *h*_1_ and *h*_2_. The thicknesses of ITO and GaAs nanoantennas are marked with *t*_1_ and *t*_2_, respectively. The optimized parameters of this absorber are set to *P* = 500 nm, *d* = 400 nm, *t*_1_ = 80 nm, *t*_2_ = 120 nm, *h*_1_ = 70 nm, and *h*_2_ = 30 nm.

The optical performances and electromagnetic field distributions of this absorber are calculated by the finite-difference time-domain (FDTD) method [43]. Periodic boundaries are employed at the *x* directions and perfect matching layers are used at the *z* directions. The dielectric constants of Ti, W, and GaAs are taken from Palik [44], and the index of ITO is 2.0 [35]. If not otherwise specified, a wide frequency plane wave with the linear polarization along the *x* axis is irradiated from the top of the nanoantenna metasurface (i.e., TM polarization) with the distance of 540 nm in between them. The transmission (*T*) in this absorber is equal to zero due to the opaque metal film used at the bottom. The absorption (*A*) of this absorber can be calculated by *A* = 1 − *R*, where *R* denotes the reflection. A finite region with the length of 20 nm, width of 500 nm, and height of 500 nm and a refined mesh of 1.6 nm are chosen to calculate the short-circuit current density (other parameters are the same with those set in the calculation of reflection). A non-uniform mesh with the minimum mesh step of 0.25 nm and the plane wave with three wavelength regions (280–400 nm, 401–1702 nm, and 1705–4400 nm) are used to calculate the standard solar spectrum using a simple two-dimensional simulation. The proposed absorber can be fabricated as the following steps: (1) orderly depositing W, GaAs, and Ti films with certain thickness on the silica substrate via the deposition method [45, 46]; (2) depositing a layer of photoresist on the structure fabricated above and etching it by the electron beam lithography [47] to form a one-dimensional nanoantenna array; (3) consecutively depositing GaAs and ITO materials with certain thickness on the structure fabricated in the second step; and (4) removing the photoresist nanoantennas coated with GaAs and ITO materials by the lift-off method.

## Results and Discussion

Figure 1b shows the absorption spectrum of the optimized absorber at normal incidence (marked with “Absorber,” black line). For comparison, the absorption spectra of the MSM structure (marked with “MSM,” red line) and the MSM structure coated only by GaAs nanoantennas (marked with “The structure without the ITO layer,” blue line) are also shown in Fig. 1b. For the structure with the simple MSM three-layer film structure, the absorption is less than 70%. When the GaAs nanoantenna period array is placed on the MSM structure, an ultra-wide absorption band with strengthened absorption from 657 to 2679 nm is achieved. This indicates that the GaAs nanoantenna array here is responsible for the strong absorption in the broad wavelength range. Note that the absorption intensities in the ranges of 991–1455 nm and 2004–2388 nm are still less than 90%. For the proposed absorber, the introduced 80-nm-thick ITO nanoantenna array further strengthens the absorption and enlarges the absorption band. Taking A > 90% into account, an ultra-wide absorption phenomenon is found with the absorption bandwidth up to 2402 nm spanning the visible, near-, and middle-infrared regions (468–2870 nm). The average absorption is increased up to 95.5%. It is because the 80-nm-thick ITO layer plays an anti-reflection role, which can further strengthen the anti-reflective effect of GaAs nanoantennas. Moreover, the 80-nm-thick ITO layer is high enough to enable a low sheet resistance, so low lateral transport losses of the carriers over hundreds of microns to lateral metallic contacts [35]. Consequentially, the great improvement on the absorption bandwidth and absorption efficiency is achieved, greater than those absorbers based on the noble metal-semiconductor composite systems [32–37]. The greatly enlarged absorption mainly originates from the excitation of GMRs and cavity modes and their hybridized coupling effects [18].

**Fig. 2:**
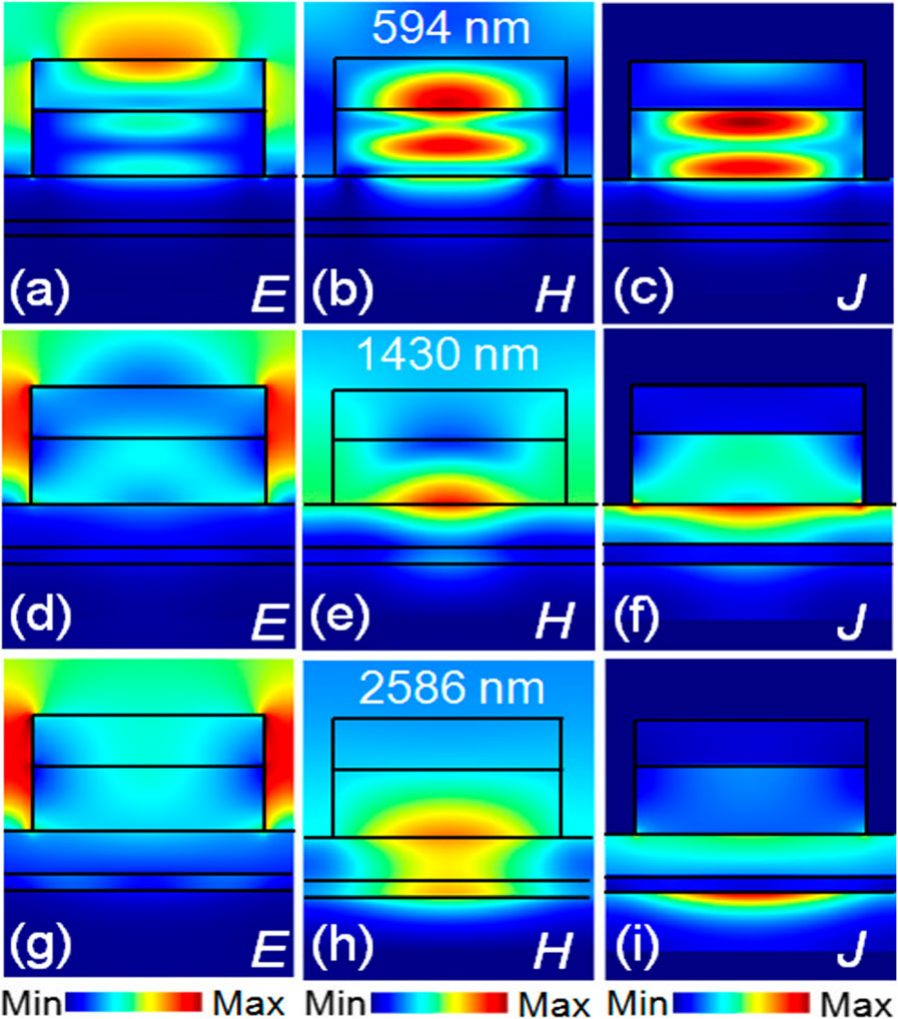
Electric field |*E*|, magnetic field |*H*| distributions, and current density *J* at 594 nm (**a**–**c**), 1430 nm (**b**–**f**), and 2586 nm (**g**–**i**), respectively

The electromagnetic field distributions (|*E*| and |*H*|) and the current density (*J*) of this absorber at different wavelength (i.e., 594 nm, 1430 nm, and 2586 nm) are investigated. At 594 nm, the electric field energy is mainly concentrated at the nanoantenna-air interface, and the strong magnetic field energy is located in the GaAs nanoantenna and ITO layer (Fig. 2a, b). These indicate the GMRs and cavity modes being excited [18, 26]. The electric current in the GaAs nanoantennas (Fig. 2c) confirms the effectiveness of GaAs nanoantennas for this absorption enhancement [48, 49]. At 1430 nm, the strong electric field mainly exists in the air slots near the nanoantennas (Fig. 2d) which implies the excited cavity modes [18, 26]. In Fig. 2e, the magnetic field energy is located at the GaAs nanoantenna-Ti film interfaces, indicating that the excited GMRs and cavity modes both contribute to the light coupled into the structure and further excite the SPPs near the interfaces of GaAs film-Ti film [9, 18, 20, 39]. The electric current distributed in the Ti film shown in Fig. 2f provides a strong evidence that the incident light is fully coupled into the structure. At 2586 nm, the electromagnetic energies mainly locate in the slots between the nanoantennas and at the interfaces of GaAs nanoantenna-Ti film and GaAs film-W film (Fig. 2g, h), and the electric current mainly distributes at the top surface of the W film (Fig 2i). These again demonstrate the light coupled into the underlying layers of the structure by the GMRs, SPPs, and cavity modes. Therefore, it is concluded that the excited GMRs, SPPs, and cavity and their synergy result in the broadband and near-perfect absorption [18].

**Fig. 3:**
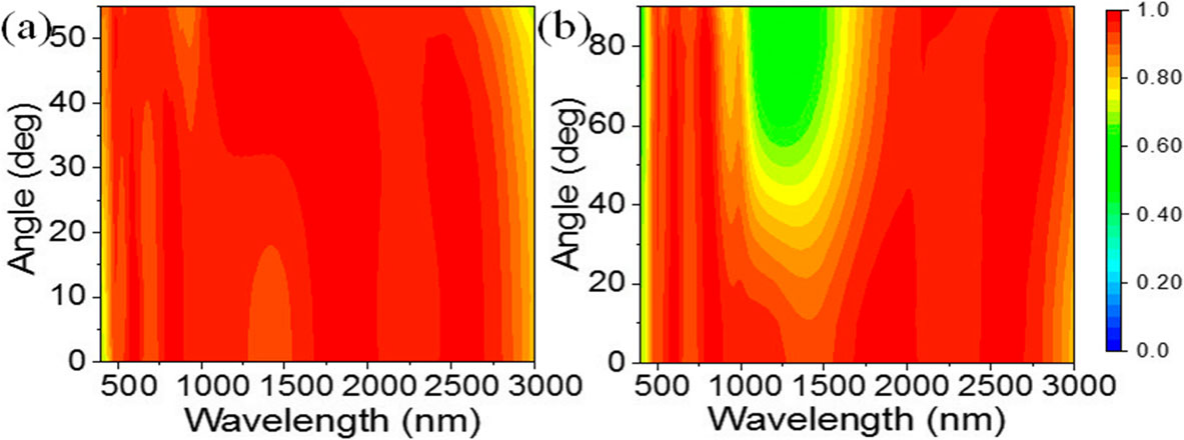
Absorption mapping of the solar absorber under a tunable incident angle (**a**) and polarization state (**b**)

In the practical applications of the solar absorbers, light absorption should be less sensitive to the incident and polarization angles [2, 3, 6, 18, 20]. However, most of absorbers based on the GaAs material seldom involve the exploration of polarization angle and incidence angle [36, 39]. Figure 3a shows the absorption evolution for the proposed solar absorber under the TM polarization with an oblique irradiation. Obviously, the absorption effect is nearly robust in the range of 468–3000 nm with the incident angle up to 55° and only a slight decrease in wavelength in the middle-infrared region. The absorption band will reduce extremely as the incident angle is over 55°. Figure 3b shows the absorption of light under different polarization states, where 0° corresponds to the TM polarization and 90° corresponds to the transverse-electric (TE) polarization. It is observed that the absorption can be maintained perfectly in the shorter and longer wavelength region (468–1010 nm and 1800–3000 nm) when the polarization angle increases from 0 to 90°. Although the absorption decreases in the near-infrared region, it is still above 50%. Overall, the angular and polarized insensitivity of the absorption should be attributed to the good matching of the impedance and the intrinsic loss [18, 19].

**Fig. 4:**
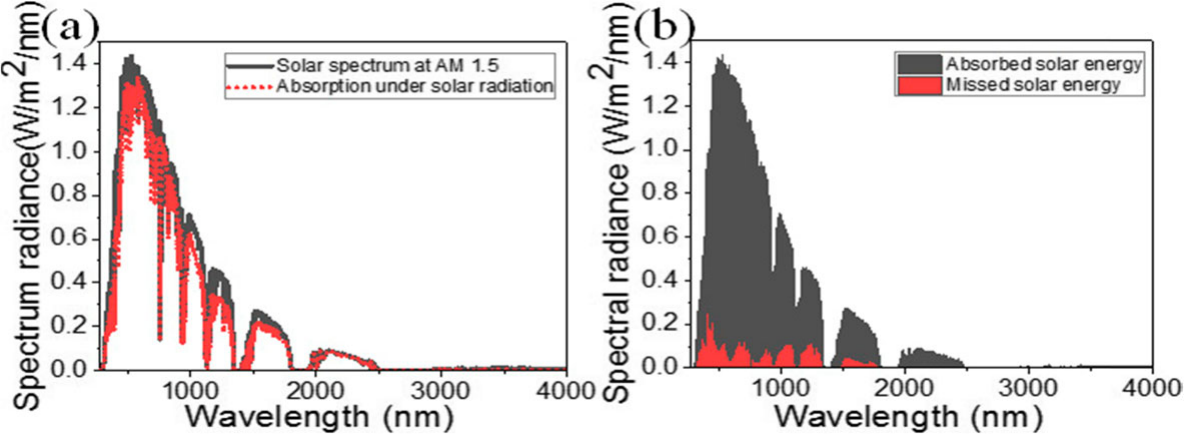
**a** Standard spectrum of solar radiance AM 1.5 and solar energy absorption spectrum of the solar absorber under the AM 1.5. **b** Absorbed and missed energy of the solar absorber in the full spectral range of solar radiance

We further carry out the solar absorption investigation by putting the optimized absorber under the illumination of AM 1.5 source. The solar absorber shows a nearly perfect absorption in the visible, near-, and middle-infrared regions, spanning the main solar irradiation energy distribution regions (Fig. 4a). Because multiple resonance states occur simultaneously, almost near-unity solar energy is captured by the absorber. These demonstrate the high solar energy absorption efficiency in such a structure. Moreover, the used refractory materials in this absorber contribute to maintaining the thermal stability of this structure when the temperature increases at a certain range. Therefore, it can be concluded that our proposed absorber has a wider application in photoelectric devices [50].

**Fig. 5:**
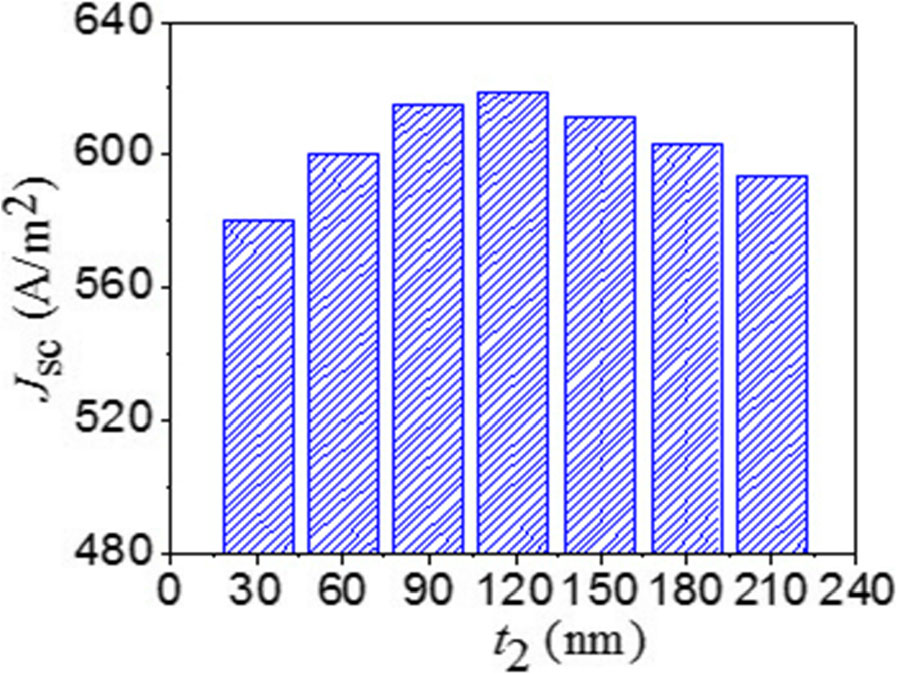
Short-circuit current density with the GaAs nanoantenna’s thickness under the TM polarized light

As reported in [36], the short-circuit current density *J*_sc_ for AM1.5 solar illumination is described by ${J}_{\mathrm{sc}}={\int}_{400\ \mathrm{nm}}^{3000\ \mathrm{nm}}\frac{e\lambda}{hc}{\Phi}_{\mathrm{AM}1.5}\left(\lambda \right)\mathrm{A}\left(\lambda \right),$ where *e* is the electron charge, *h* is the Planck constant, *λ* is the light wavelength, Φ_AM1.5_(λ) is the solar radiance at AM 1.5, A(*λ*) is the absorption, and *c* is the light speed. Here, we investigated the short-circuit current density by changing the thickness of GaAs nanoantennas with other parameters invariable. When *t*_2_ is tuned from 30 to 210 nm with a step of 30 nm, the collected photocurrent is derived as shown in Fig. 5. A strong regularity with the thickness *t*_*2*_ is obtained because *J*_sc_ mainly relies on the number of resonant modes in the range of 300–3000 nm. The maximum *J*_sc_ equal to 61.947 mA/cm^2^ is obtained when *t*_2_ = 120 nm, which is much larger than that reported by Meng et al. (30.3 mA/cm^2^) [35].

## Conclusion

We present a solar absorber based on the GaAs nanoantennas covered by a single layer ITO on a thin W-GaAs-Ti three-layer stack structure. An ultra-broadband near-perfect absorber is achieved in the wavelength range of 468–2870 nm with the average absorption over 95%. The ultra-broadband absorption property originates from the synergy of GMRs, cavity modes, and SPPs. The ultra-broadband solar perfect absorber also has great tolerance for temperature, insensitivity to the angle and polarization of incident light, and best short-circuit current density up to 61.947 mA/cm^2^. These offer new perspectives for achieving thin film solar cells, solar energy harvesting, and thermal emitters.

## Data Availability

All data generated or analyzed during this study are included in this article.

## Abbreviations

TM: Transverse-magnetic
1D: One-dimensional
AR: Antireflection
GMRs: Guide mode resonances
SPPs: Surface plasmon polaritons
MSM: Metal-semiconductor-metal
FDTD: Finite-difference time-domain
TE: Transverse-electric
